# Testing sex and gender in sports; reinventing, reimagining and reconstructing histories

**DOI:** 10.1016/j.endeavour.2010.09.005

**Published:** 2010-12

**Authors:** Vanessa Heggie

**Affiliations:** Department of History & Philosophy of Science, University of Cambridge, Free School Lane, Cambridge, Cambridgeshire CB2 3RH, United Kingdom

## Abstract

Most international sports organisations work on the premise that human beings come in one of two genders: male or female. Consequently, all athletes, including intersex and transgender individuals, must be assigned to compete in one or other category. Since the 1930s (not, as is popularly suggested, the 1960s) these organisations have relied on scientific and medical professionals to provide an ‘objective’ judgement of an athlete's eligibility to compete in women's national and international sporting events. The changing nature of these judgements reflects a great deal about our cultural, social and national prejudices, while the matter of testing itself has become a site of conflict for feminists and human rights activists. Because of the sensitive nature of this subject, histories of sex testing are difficult to write and research; this has lead to the repetition of inaccurate information and false assertions about gender fraud, particularly in relation to the ‘classic’ cases of Stella Walsh and Heinrich/Hermann/Dora Ratjen. As historians, we need to be extremely careful to differentiate between mythologies and histories.

## The athletic girl not unfeminine^1^

The relationship between femininity and physical exercise is well studied by historians; in fact, it is probably better analysed and certainly more problematised than the relationship between masculinity and sport.^2^ It comes as no surprise that as major international sporting events were developed in the very late nineteenth century issues of physical display, modesty, muscularity, competition and the perpetual risk of sterility were all used to exclude women from many sporting activities. Virtually the only way women could participate in competitive sport was through sexually segregated events.

The standard account of this process is as follows: the early years of the twentieth century are generally represented as a period of struggle and triumph for women's sports, with the eventual acceptance of a significant women's programme in the Olympics in 1924 and the proliferation of national organisations for women's sport.^3^ But as international sport took its place as ‘sublimated war’ at mid-century, the desire to win apparently became so pressing for some nations that deliberate and systematic cheating took place in both the men's and women's events. Consequently, doping and gender fraud became central concerns in the late 1950s and 60s, resulting in the eventual introduction of systematic testing for both at international sporting events in the late 1960s. The usual explanation given for the introduction of sex testing is a list of gender frauds (or suspected frauds) most of whom fulfil a specific stereotype: physically muscular, deep-voiced competitors living under totalitarian, fascist or communist regimes. Amongst the ‘canon’ of gender frauds are normally the well-built and extremely successful Ukrainian sisters, Irina and Tamara Press, who dropped out of international competition when gender tests were introduced.^4^ For an earlier example, the usual name listed is Hermann Ratjen, who, we are told, through loyalty to the Hitler Youth, bound his genitals and competed as a woman – Dora Ratjen – at the 1936 Berlin Games. Ratjen may have been discovered as a fraud at a sporting event in the late 1930s, and was definitively revealed by a journalist in the 1950s, to whom he told his story.^5^ Ratjen's case has even been made into a feature-length, award-winning movie.

There are three problems with this account. Firstly, it ignores the fact that systematic sex testing, of a sort, existed at least as early as the 1940s. Secondly it obscures the fact that the first well-known gender ‘frauds’ in international sport were not Nazi sympathisers, or Communist state athletes, but a British shot putter, and a Czechoslovakian runner. Finally, Hermann Ratjen's name was not Hermann, but Heinrich, and documents (including medical records) released after his death in 2008 suggest that rather than being a conspiracy, his place on the 1936 German women's high-jump team is explained by a more mundane and human case of gender uncertainty, medical error, fear and embarrassment.

The story of sex testing, and *histories* of sex testing, in international sport tell us a great deal about social attitudes to gender, and how the co-option of science in sport (however it is resisted by scientists and human rights campaigners) can act to essentialize social categories.^6^ Sex testing, after all, is a tautological (or at least circular) process: the activities which we recognise as sports are overwhelmingly those which favour a physiology which we consider ‘masculine’. As a general rule, the competitor who is taller, has a higher muscle-to-fat ratio, and the larger heart and lungs (plus some other cardio-respiratory factors) will have the sporting advantage. It is therefore inevitable that any woman who is good at sport will tend to demonstrate a more ‘masculine’ physique than women who are not good at sport. What the sex test effectively does, therefore, is provide an upper limit for women's sporting performance; there is a point at which your masculine-style body is declared ‘too masculine’, and you are disqualified, regardless of your personal gender identity. For men there is no equivalent upper *physiological* limit – no kind of genetic, or hormonal, or physiological advantage is tested for, even if these would give a ‘super masculine’ athlete a distinct advantage over the merely very athletic ‘normal’ male. (Of course, both men and women are liable to the same testing regimes when it comes to external sources of advantage, i.e. doping.) There are probably hundreds of genetic variations which lead to ‘unfair’ advantages in sport; only those associated with gender are used to exclude or disqualify athletes.

This article will explore how this unequal situation came about, and discuss in brief the various technologies of gender and sex testing in sports. ‘Gender’, ‘sex’ and ‘femininity’ are often used interchangeably when referring to testing in sports despite having quite different meanings; in this article I will quote directly from the primary sources or use the phrase ‘sex testing’.^7^ More importantly I will also attempt to disrupt the comfortable narratives we have in place of sex-frauds being over-competitive athletes from non-democratic regimes.^8^

## The canon of gender frauds

There is a straightforward narrative path through the history of gender testing, which takes as its earliest starting point the Berlin Olympics of 1936. This is where Ratjen competed as a woman in the high-jump, but our attention is more often directed at the 100 m race. Here the American runner Helen Stephens (the ‘Fulton Flash’) was accused of being a man when she narrowly beat the favourite, Polish runner Stanisława Walasiewicz. Stephens underwent an unspecified test and was declared a woman by the Berlin authorities, taking gold.^9^ Nearly four and a half decades later, in 1980, Walasiewicz (now known by her Anglicized name, Stella Walsh, which will be used through the rest of this article) was shot dead in a department store car park in Ohio. She had emigrated to the USA in 1947, and retired from competitive sport in 1951, continuing to do charitable work for young athletes. As her death was a violent one, the consequence of an armed robbery, her body was autopsied. At this point ‘ambiguous’ sexual features were made public.

The revelation of an Eastern European ‘sex fraud’ came at a sensitive time for international sports, as the Summer Olympics that year had been held, controversially, in Moscow. Despite demands that her medals and accolades should be revoked, the International Olympic Committee (hence: IOC) finally issued a statement saying that Walsh had competed in good faith, and had not broken the rules of the day.^10^ Nonetheless Walsh has joined Ratjen in the canon of gender frauds, as the poster girl and poster boy for ‘sex cheats’. They occur in almost every historical account, text book of sports medicine, or newspaper article about the latest successful female athlete whose physique has provoked suspicion. Both Walsh and Ratjen fit the conventional narrative about the history of sex testing; one a tool of a fascist regime, the other Eastern European, if not strictly a Communist athlete. In Walsh's case the ‘irony’ of the accusation against the all-American Stephens makes her story irresistible to most authors, whether they’re journalists, historians or scientists.

Of course, neither Walsh nor Ratjen had anything whatsoever to do with the introduction of sex testing. Both their stories were revealed years, in one case nearly thirty years, after they had stopped competing, and neither were made broadly public until after systematic sex testing had already been introduced into international sports. Shortly after the accusation that Stephens was a man her team coach, Avery Brundage, called for more systematic screening of suspicious cases. But when he chose to point the finger at ‘suspicious’ cases, it was neither Ratjen nor Walsh that he named. Instead Brundage named the Czechoslovakian runner Zdenka Koubkova who had changed sex from female to male (becoming Zdenek Koubek) in 1936, and the British shot putter and javelin thrower Mary/Mark Weston. Ratjen and Walsh's stories have been reinvented to fit narratives about the Cold War and the politicisation of sport; in so doing, the stories of Koubek and Weston have been lost.

## The Devonshire Wonder

Mary Louise Edith Weston (b. ∼1906) competed nationally for the Middlesex Ladies’ Athletics Club and internationally with the British Olympic Team earning the nickname ‘the Devonshire Wonder’ due to exceptional performances in the javelin and shot put. Weston took the Women's Amateur Athletic Association shot put title in 1925 and 1928, winning all three throwing events (shot, javelin and discus) in 1929. Weston also won the international women's shot put title in 1934.^11^ Shortly after this achievement, Mary Weston had ‘a series of operations in Charing Cross hospital’, after which he abandoned competitive sport to take up a career as a masseur, and changed his name to Mark.^12^

**Figure 1:**
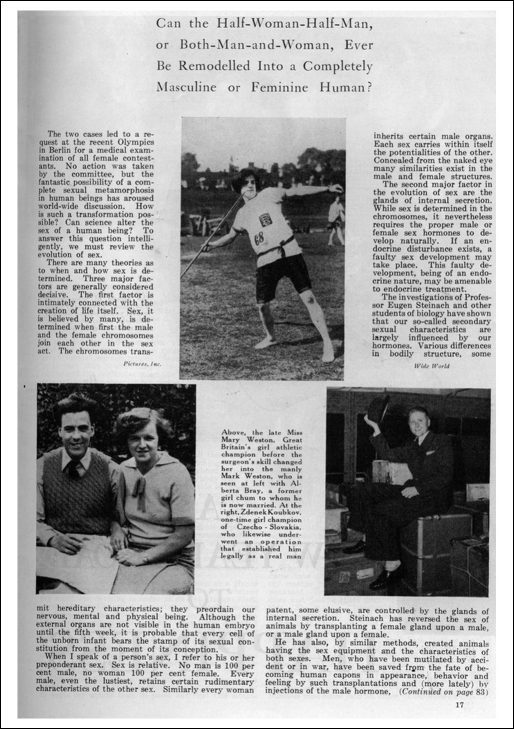
A page from an article in *Physical Culture* about transsexual individuals in sport. Pictured from left to right are Mark Weston and his partner; Mark Weston competing as Mary Weston; and Zdenek Koubek. D.F. Wickets, Can Sex in Humans Be Changed, *Physical Culture* (January 1937) pp. 16–7 & 83–5. Unsuccessful attempts were made to trace the rights holders to this image. See also note 11.

Weston's story gained media attention in the USA as well as the UK, with close coverage in the British *Daily Mirror*, and special features in North American in *Physical Culture* and *Time*. It's noticeable how positive, generally, this coverage is (especially compared to later media treatment of ‘unfeminine’ athletes). The *Daily Mirror* carefully pointed out that Weston had won the international shot putting title while unaware that he was ‘competing unfairly against other women competitors’ – it was an honest mistake, not a case of fraud.^13^ Similar sentiments were repeated whenever Weston made the news; in 1938 when he married his long-term best friend Miss Alberta Bray, a ‘shy blonde in her early twenties’, and tragically in 1942 when his older brother Harry (previously known as ‘Hilda’), hung himself after becoming ‘depressed following [his] operations for change of sex’ (see Figure 1).^14^

Zdenek Koubek's story was similar: he had set national records for long jump, high jump and some track events in Czechoslovakia, and a women's world record for the 800 m in 1934, before requesting in 1935 or 36 that the state should recognise him as a man. He retired from sport to pursue a career in cabaret, which took him to the USA, where he was interviewed by *Time* magazine which ran two stories relating to sex fraud and the 1936 Berlin Olympics. These articles discussed the fears of ‘worry-ridden Avery Brundage’ who ‘demanded examination for sex ambiguities in all women competitors’.^15^ Brundage was not the only one advocating that segregation in sports needed to be more vigorously policed, and it was suggested at this time that sex tests should become compulsory at the next Olympic Games, due to take place in 1940. Although the Second World War disrupted the Olympic schedule international sports organisations rapidly took up the suggestion that some legislation on gender ought to be introduced. By 1946 the International Amateur Athletics Federation (hence: IAAF) had introduced a rule requiring female competitors to bring a medical certificate to prove they were eligible to compete (IAAF rule 17 paragraph 3). From 1948 (London), the IOC also required female competitors to comply with the IAAF's requirements.^16^ So, it is clear that this concern about gender fraud is a phenomenon of the 1930s and ‘40s, and not solely one of the 1950s or ‘60s. That said, although certificates were required, these were not evidence of a standardized, internationally recognized gender test. Since neither the IOC nor the IAAF actually defined ‘femininity’ the assumption was that the social or cultural definition in any nation was acceptable for sports, and that any nation's judgement could be trusted. It is this that changed in the 1960s.

## Standardizing femininity

The dramatic successes of female athletes from the USSR and GDR in the middle decades of the twentieth century certainly caused the sports organisations of the West some concern. That some of these women had physiques which transgressed traditional Western notions of femininity was obvious, and other historians have written about the ways in which appropriate and inappropriate physicality were discussed in the popular media. Epitomising this trend were Irina (1939–2004) and Tamara (b.1937) Press, sisters from Ukraine who competed in hurdles and pentathlon, and shot put and discuss respectively. The decision of the Press sisters, along with several of their co-competitors, to drop out of international sport coincidental with the introduction of formal sex tests only added fuel to the rumours about their gender. That the sex tests were necessary seemed to be confirmed by the absence of ‘suspicious’ athletes after their introduction. (The fact that two British athletes in the 1960 Rome Olympic team were accused by some newspapers of being men seems rarely to be reported by historians).^17^

So systematic, at-event, standardized, ‘scientific’ sex tests were introduced in the 1960s because the process of femininity certification by team and family doctors that already existed could no longer be trusted. The first tests took place at the 1966 European Athletics Championship in Budapest, where female athletes were asked to undergo a visual examination of the genitals and secondary sexual features, carried out by a panel of three female doctors.^18^ Other sports events instigated similar investigations, and a ‘naked parade’ was used at the 1967 Pan-American Games in Winnipeg. Even more invasive approaches were also trialled; at the 1966 Commonwealth Games in Jamaica female athletes were challenged by a manual examination, likened by one athlete to ‘a grope’.^19^ Unsurprisingly, these tests were deeply unpopular with many athletes, who saw them as invasive, and often functionless or unfair – as the American shot putter Maren Sidler said of the tests in Winnepeg:

They lined us up outside a room where there were three doctors sitting in a row behind desks. You had to go in and pull up your shirt and push down your pants. Then they just looked while you waited for them to confer and decide if you were OK. While I was in line I remember one of the sprinters, a tiny, skinny girl, came out shaking her head back and forth saying. ‘Well, I failed, I didn’t have enough up top. They say I can’t run and I have to go home because I’m not ‘big’ enough.’^20^

Not only were the tests perceived as crude and unpleasant, they were also not sufficient in and of themselves. Although it is not generally noted, the first person to be formally disqualified from women's sports (as opposed to the informal discouragement of girls who were ‘not big enough’) was only excluded after a *series* of tests. Ewa Kłobukowska (b.1946), a Polish sprinter who had passed the gender test at Budapest in 1966, caused concern at the 1967 European Cup Track and Field Event in Kiev. After failing a ‘close-up visual inspection of the genitalia [which] was used to establish eligibility’ she underwent further testing.^21^ This included a prototype chromosomal test, which Kłobukowska failed. This chromosomal test (sometimes imprecisely called a ‘genetic’ test) was the Barr body test, and was adopted by the IOC in 1967, who trialled it at the 1968 Winter Games in Grenoble. The trial led to the disqualification of the Austrian downhill skier then known as Erica Schinegger (who had sex reassignment surgery and lived subsequently as Erik) and was rolled out using a lottery system for the Summer Games that year held in Mexico City. A reader of the Official Report from the Mexico City Games will look in vain for specific references to sex testing, however; despite the fact that the IOC's leading expert in sex testing, Dr Eduardo Hay, was a professor of obstetrics and gynaecology from Mexico, there is no mention of the testing done on female competitors, although other work was carried out by the ‘laboratory for human biological and genetic research’.^22^

The test the IOC chose was the Barr Body test, which involves screening cells taken from the inside of the cheek (a buccal smear). Barr Bodies are cellular artefacts that are easily stained and visualised under a microscope, and a positive test was taken – for the purposes of sports sex testing – to indicate that a cell's sex chromosome complement is XX rather than XY. The Barr Body itself is an inactivated X chromosome; since only one X chromosome is necessary for biological function (otherwise XY human beings would be fatally compromised) the ‘spare’ chromosome folds in on itself forming a dense chromatin body. As critics, both at the time and subsequently, have pointed out, this test for chromosomal sex does not necessarily map on to physiological or phenotypic sex, which are the only kinds of sexual identity that confer a sporting advantage (and there are many confounding conditions, as people can be born with just one or three or more sex chromosomes, so that combinations like XXY or XO are quite possible).^23^ Nonetheless this was the test used at the Olympic Games through the 1970s and into the 1980s, and although thousands of athletes were tested, none were (officially) reported to have failed after 1968. This low detection rate may, of course, be due to the fact that home nations could easily test their athletes themselves – in the UK the British Association of Sport and (Exercise) Medicine made a ‘sex test’ and a ‘chromatin count service’, available to British governing bodies of sport in 1970.^24^

**Figure 2:**
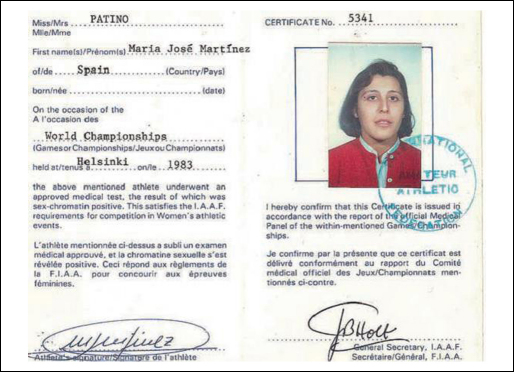
Mario Patino's ID Card.

In the mid-1980s the high profile case of Spanish hurdler Maria Martinez-Patino, who fought a three-year campaign for reinstatement after being disqualified, was used to pressure the IOC and other organisations into changing (or eliminating) their sex tests. Patino failed a Barr Body test at the World University Games held in Kobe, Japan, in 1985, and was instructed by her coach to retire from sport with an ‘injury’; she refused to do so, and when she started competing in Spain again she was formally disqualified and had her medals and records revoked.^25^ Patino eventually succeeded in overturning the ruling, based on the principle that a specific medical condition (androgen insensitivity syndrome) meant that she ‘failed’ a Barr body test, while gaining no physiological sporting advantage – so the argument was not that testing for sex was problematic in and of itself, but rather than this specific test, using chromosomes as a proxy for sporting ability, was inappropriate. This successful appeal was almost certainly due to the fact that human rights activists and geneticists who did not believe the test was fair took up her cause as a test case through which they could make their points about equality, scientific objectivity, and the complexity of human gender identity (see Figure 2).

The IAAF was the first to drop the sex testing requirement for international competitions (somewhat ironically, as it was also the first organisation to introduce femininity checks via its requirement for certificates in the 1940s, as discussed above). In 1988 it dropped chromosomal and genetic testing in favour of a manual/visual ‘health check’ by the team doctor, and then abandoned all forms of systematic sex testing in 1992. The IAAF argued that these were no longer necessary because doping regulations required athletes to pass urine in front of witnesses, and that modern sportswear was now so revealing that it seemed unfeasible that a man could masquerade as a woman.

The IOC was more resistant to change, instead introducing a new sort of test in 1992, a genetic test which identifies a specific region of code usually found on the Y chromosome and known as the ‘sex determining region Y’. It was considered that the presence or absence of this single gene (which in turn controls the expression of another gene that codes for a protein vital to testicular formation) was a better marker of gender than the presence of X or Y chromosomes.^26^ Even this test continued to throw what, for the purposes of sex segregation, seem to have been false positives, as although in Atlanta in 1996 eight women ‘failed’ this test all were allowed to compete after further examinations were carried out.

Finally, in 1999 the IOC agreed to follow the IAAF and remove the requirement for blanket sex testing, so that the Millennium Games in Sydney, 2000, were the first Games in three decades where the genetic make-up of female athletes were not scrutinised. Of course, as we have seen recently in the case of Caster Semenya, if the gender of an athlete is actually challenged, she can still be required to undergo a full gamut of tests: physiological, genetic, hormonal, psychological.

## Ratjen revisited: reinventing gender fraud

For nearly a year, from her success in the 800 m at the World Championships in Athletics, August 2009, to the final declaration of her femininity from the IAAF in July 2010, the story of Caster Semenya has provoked journalists and academics to explore the history of sex testing in international sports. In the process these stories have helped to consolidate a mythology of gender fraud, which has not been shaken even by the revelation of new archive materials. In fact, this coverage has led to what I believe is a brand new myth about gender fraud, this time reinventing Stella Walsh as a ‘suspect’ athlete in the 1930s, when in fact her femininity was (comparatively) unproblematic until her death in 1980.

Gender is an extraordinarily difficult topic for historical analysis; it is an intrinsically embodied, lived experience which we can find hard to reconstruct, and it is also a personal and private matter which is often deliberately concealed from official records, both medical and sporting. By relying instead on a closed ‘canon’ of publically available information, often newspaper reports and early articles about sex testing, writers, including historians (and including myself), have restated and reinforced stories about gender fraud which are not based on first-hand primary material or archive research. Originally, and most obviously, the strong association between the tensions of the Cold War and sex testing has obscured the stories of earlier transsexual athletes which do not fit the pattern of post-war gender ‘frauds’.

But more worryingly, as some stories have been lost, others have explicitly been reinvented or re-imagined to fit this Cold War storyline. Stella Walsh's story is such a case, and is one that is currently in the active process of being re-created to fit with the narrative of gender testing which starts with the ‘Press Brothers’, and in which gender frauds are explicitly ‘other’ to the white western world: communist, fascist, Black. Walsh's reinvention started at least as early as the 1990s, as the following quote, from a paper in the *Journal of the Medical Association of Georgia* demonstrates:

In the 1932 Olympic games the 100-m sprint champion was found to have testes during a 1980 autopsy after her accidental death.^27^

While this quote gets the dates right, the grammatical strangeness of it seems to suggest that Walsh's gender was suspect in 1932, which of course it was not (this is not even a mis-reference to the Games where accusations of gender fraud were leveled at Stephens for beating Walsh, as that was in 1936).

More recently the case has been explicitly made that Walsh was a suspected fraud as early as the 1930s. In its obituary of Irina Press on 31 May 2004, the UK newspaper the *Telegraph* claimed that Walsh was one of the athletes who, like the Press sisters, had incited speculation about gender. Suggesting that Walsh was ‘a man’ the paper claims that before the Press sisters retired from sport

[t]here had been several scandals of the kind, notably that of Stella Walsh, who won the 100-yard dash at the 1932 Olympics. Her rivals believed that she was a man, and in 1980 an autopsy revealed they were right.^28^

This reinvention of Walsh's story, so that it is a ‘scandal’ of the 1930s, has not only reached into the academic and medical journals, but along the way has gathered a ‘legend’ (in both senses of the word).

A recent article in the *South African Journal of Sports Medicine* repeats the suggestion that Walsh's gender was a subject of public questioning in the 1930s, and claims that newspapers referred to her as ‘Stella the Fella’.^29^ The reference for this claim is an article in the *Journal of the Royal Society of Medicine*, which does not in turn give a source for its belief that not only was Walsh's gender suspect and that she generated ‘Stella the Fella’ headlines, but also that it was actually Walsh who personally challenged Stephen's eligibility in 1936 (most earlier accounts have a Polish journalist as the accuser).^30^ The claim that there were contemporary ‘Stella the Fella’ headlines is even repeated in sociology texts.^31^ But such reports of specific, printed suspicions about Walsh's gender in the 1930s would seem to have appeared in articles only since 2004, i.e. after the claim was made in the *Telegraph* obituary.

It is of course possible that Walsh's gender was questioned in some circles, and her manly physique was certainly *noted* in newspaper articles. But Helen Stephen's strapping figure was also commented on, and the bodies of two British athletes were athletic enough to have them accused of being men in 1960 (see above); there are dozens (probably hundreds) of other cases where the successful, physically powerful woman athlete has been subject to suspicions, rumour and innuendo. If we selectively remember only rumours that were ‘revealed to be right’, suspicion would appear a disproportionately powerful predictor of gender ambiguity. This is a dangerous ‘lesson from history’ if it is to be applied to female athletes today.

**Figure 3:**
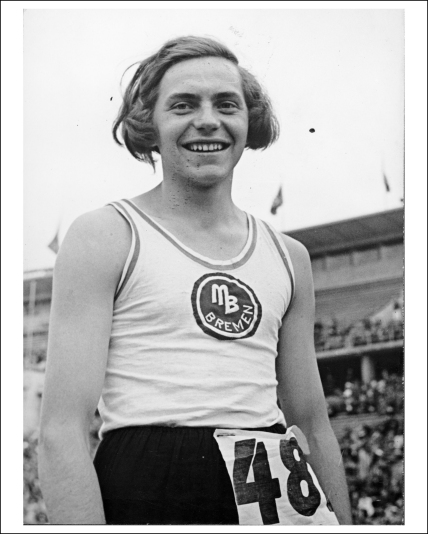
Heinrich Ratjen competing as ‘Dora’ at a track and field event in Berlin in 1937. Bild-183C10336. Reproduced with permission from the The Digital Picture Archive of the Press- and Information Office of the [German] Federal Government: http://www.bundesbildstelle.de/.

**Figure 4:**
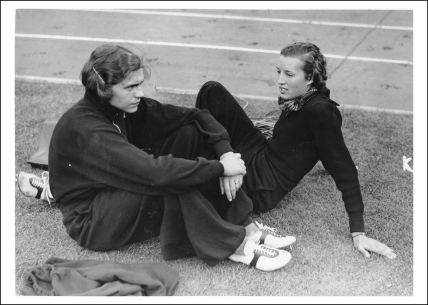
Heinrich Ratjen and another high-jump team member, Elfriede Kaun, in 1937. Bild-183C10336: Reproduced with permission from the The Digital Picture Archive of the Press- and Information Office of the [German] Federal Government: http://www.bundesbildstelle.de/.

Even more recent, however, is the revisitation of Heinrich Ratjen (still often called ‘Hermann’). Ratjen, after all, is the archetypal story of gender fraud – to date in fact the only ‘genuine’ case of a man masquerading as a woman. The movie, *Berlin 36*, which was released in Germany in the Autumn of 2009 dramatises Ratjen's case, using the pseudonym ‘Marie Ketteler’ in order tell the story of another excluded athlete, Gretel Bergmann. Bergmann was a Jewish athlete, and her exclusion from the German Olympic team (ostensibly on grounds of underperformance) was almost certainly because of her ethnicity. She was replaced by Ratjen, which again fulfils a neat narrative of the totally corrupt, transgressive fascist body and sport (see Figures 3 and 4).

But in response to the film, an investigative journalist from *Der Spiegel* pursued the question of Ratjen's gender, and retrieved original material from the Department for Sexual Medicine at Kiel University Hospital, which reinterprets Ratjen's story in a way entirely consistent with contemporary gender controversies, and which undermines our typical story of fascist/communist transgression. According to these records, Ratjen's gender ambiguity was not discovered at a sporting event, or revealed to a journalist in the 1950s, but was due to an ID-card challenge made by a German police officer at a train station. While Ratjen's identity card said he was female, the police officer believed him to be male; a medical examination declared him a man. Although the Reich Sports Ministry objected, and requested further tests and a stay in a sanatorium, eventually Ratjen was accepted as a man, officially re-designated, and given a new name and papers. Ratjen's circumstances appear to have been a consequence of confusion shortly after his birth, which neither he nor his parents seemed able to rectify when his identity became conflicted during adolescence, after a childhood raised unquestioningly as a girl. Ratjen was after all only 17 in 1936 when asked to compete for the Fatherland. Although the story of deliberate Nazi fraud makes better headlines, Ratjen's story is probably a more homely and familiar one of medical error, gender uncertainty, and embarrassed silences.^32^

## Reconstructing gender

There is a difference between history and the *uses* of history. Without doubt the media coverage of gender frauds has laid them into particular narrative patterns. They are often stories of direct conflicts between good and evil, the wonders of modern science in a battle against frauds, or the dangers of a medicalised, over-competitive sports ideology where even gender identity and sexual health can be sacrificed in the cause of national sporting success. There is considerable slippage between anti-drug rhetoric and discussions of sex testing – indeed, in some cases there is clear confusion between the androgenisation caused by steroids and conditions which might cause a woman to fail a sex test (it is unlikely that any sex test, now or in the past, would fail a competitor merely for the side-effects of steroid use). However, in most sources in the popular media, and many in the medical and scientific press, there is little critique of the fundamental concept that there *are* two types of athlete – and only two types – who can in theory be objectively, scientifically distinguished. (The question of whether genders, even if they exist, *should* be distinguished, or whether other measures would better ensure fairness in sport is usually left to academic work in philosophy and sociology.) It is therefore also the case that stories of gender fraud can reinforce a range of cultural and social understandings of femininity.

So we need to be careful about how we participate in the reinvention and reimagining of history; while the representational politics of gender testing are worth exploring, there is a risk that unless carefully written, our accounts of the *representation* of Ratjen or Walsh can be used by other writers to *reinvent* these historical actors’ stories (e.g. causing a newspaper's printed suspicions to become the lived reality). At the same time, we have clearly under-written other aspects of gender testing. There are very few articles by historians on the development of the technology of sex testing for sports, or on the conscientious objectors, the scientists who refused to take part and who advised and campaigned against the use of tests. There is no account explaining why the IOC chose the Barr Body test in the 1960s, or the sex determining region Y test in the 1990s. There is no thorough explanation for the difference between the IAAF and the IOC in the 1990s (and little to nothing on other sporting organisations’ gender testing).

Finally, we do not have a good account of sex testing and gender uncertainty before the Cold War. Athletes such as Weston are virtually written out of the current story, but need to be understood in their own context – i.e. within the revived fascination with sexual differentiation, hormones, steroids and organ therapy of the 1920s and ‘30s.^33^ Cultural fears that sport and exercise might be medically dangerous to women are well studied, but when exactly did concerns that exercise might virilise women turn into a fear that being virile *and* being a woman was ‘cheating’?

## Figures and Tables

Taken from: M Martinez Patino, Personal Account: A Bild 183-C10379: woman: tried and tested, *Lancet***366** (2005 – supplement) p. 38–9.

## Footnotes

1 The quote comes from a title in an American sports magazine: C.T. Herrick, Women in Athletics: The Athletic Girl Not Unfeminine, *Outing***40** (1902), pp. 713–21.

2 For a good starting point, see Patricia Vertinsky's article & bibliography: P. Vertinisky, Gender Relations, Women's History and Sport History: A Decade of Changing Enquiry, 1983-1993, *Journal of Sport History***21** (1994), pp. 1–24 & 25–8.

3 A. Jutel, ‘Thou dost run as in flotation’: femininity, reassurance and the emergence of the Women's marathon, *International Journal of the History of Sport***20** (2003), pp. 17–36; G.P. Moon, A New Dawn Rising; An Empirical and Social Study concerning the emergence and development of English Women's athletics until 1960, PhD, Roehampton Institute (1997); J. Hargreaves, Sporting Females: Critical Issues in the History and Sociology of Women's Sport, Routledge, London (1994); A. Guttmann, Women's Sports*:* A History, Columbia University Press, Columbia (1992). For a slightly different interpretation which emphasises the continuation of paternalistic control see F. Carpentier et al., The Modern Olympic Movement, women's sport and the social order during the inter-war period, *International Journal of the History of Sport***23** (2006), pp. 1112–7.

4 B.D. Dickinson et al., Gender Verification of Female Olympic Athletes, *Medicine & Science in Sports & Exercise*, **34** (2002), p. 1539–42; L.J. Elsas, R.P. Hayes, K. Muralidharan, Gender Verification At The Centennial Olympic Games, *The Journal of the Medical Association of Georgia***86** (1997), pp. 50–4; T. Todd, ‘Anabolic Steroids and Sport’, in J Berryman and R Park (eds.), Sport and Exercise Science: Essays in the History of Sports Medicine (Chicago, 1992), pp. 319–50; E.A. Ferris, Gender Verification Testing in Sport, *British Medical Bulletin***48** (1992), pp. 1–15 and of course J. Hoberman, Mortal Engines: The Science of Performance and the Dehumanization of Sport, Blackburn Press, New Jersey (1992).

5 Anon, High Jump She is a He – So Dorothy gets a World Record, *Daily Mirror* (24 Jul. 1957), p. 3.

6 For further critiques on drug and gender testing see: L. Wackwitz, Verifying the Myth: Olympic Sex Testing and the Category ‘Woman’, *Women's Studies International Forum***26** (2003), pp. 553–60; I.E. Ritchie, Sex tested, Gender verified: controlling female sexuality in the age of containment, *Sports History Review***34** (2003), pp. 80–93; L.R. Davis & L.C. Delano, Fixing the Boundaries of Physical Gender: Side Effects of Anti-Drug Campaigns on Athletics, *Sociology of Sport Journal***9** (1992), pp. 1–19.

7 For a good introduction to the science of sex testing, which can only be briefly discussed here, see Dickenson et al. (2002).

8 On science and sport also A. Wrynn, The Human Factor: Science, Medicine and the International Olympic Committee, 1900–70, *Sport in Society***2** (2004), pp. 211–31.

9 A. Carlson, Essay: Suspect sex, *The Lancet***366** (2005 – supplement) pp. 39–40.

10 E. Hay, The Stella Walsh Case, *Olympic Review***162** (1981), pp. 221–2; D. Langlais, The Road Not Taken: the sex secret that really didn’t matter, *Running Times*, Oct. 1988.

11 D.F. Wickets, Can Sex in Humans Be Changed, *Physical Culture* (January 1937) pp. 16–7 & 83–5. *Physical Culture* back copies have been digitised and made available by the Digital Media Repository at Ball State University, USA – see http://libx.bsu.edu/ [accessed August 2010]. Stories from *Physical Culture* and other popular science/health magazines are also posted on the Modern Mechanix blog, where I first encountered Mark Weston: http://blog.modernmechanix.com/ [accessed August 2010].

12 Anon, She's Now A Man, *Daily Mirror* (29 May 1936), p. 6.

13 Anon, Woman Athlete Who Became A Man Married to ‘Girl in a Million’, *Daily Mirror* (11 Aug. 1938), p. 1.

14 Anon, 2 Sisters Became Brothers, *Daily Mirror* (20 Jul. 1942), p. 4.

15 Olympic Games, *Time* (10 Aug. 1936); Change of Sex, *Time* (24 Aug. 1936), both available via http://www.time.com/time/magazine [accessed July 2010].

16 Moon (1997), p. 293.

17 Anon, Olympic Women ‘have medical certificates’, *Times* (22 Aug. 1960), p. 4.

18 L.J. Elsas et al., Gender Verification of Female Athletes, *Genetics in Medicine***2** (2000), pp. 249–54.

19 S. Lynch, When men were men…and so were the women, *Guardian* (7 Aug. 2004), p. 82.

20 D. Larned, The Femininity Test: A woman's first Olympic hurdle, *Womensports***3** (1976) pp. 8–11: p. 8.

21 Anon, Girl Athlete To Have New Sex Tests, *Daily Mirror* (20 Sept. 1967), p. 6.

22 All the Official Reports for the modern Olympic Games are available via: http://www.la84foundation.org/5va/reports_frmst.htm.

23 A. de la Chapelle, The Use and Misuse of Sex Chromatin Screening for ‘Gender Identification’ of Female Athletes, *Journal of the American Medical Association***256** (1986), pp. 1920–23.

24 Archives of the British Association of Sport and Exercise Medicine, Minutes of the Executive Committee 7 March 1970. The archives of the BAS(E)M have been deposited at the Wellcome Library under the classmark SA/BSM. I would like to thank Dr Neil Carter for providing me with copies prior to their deposition.

25 M Martinez Patino, Personal Account: A woman tried and tested, *Lancet***366** (2005 – supplement) pp. 38–9.

26 B. Dingeon, Gender verification and the Next Olympic Games, *Journal of the American Medical Association***269** (1993), pp. 357–8.

27 L.J. Elsas et al., Gender Verification At The Centennial Olympic Games, *The Journal of the Medical Association of Georgia***86** (1997), pp. 50–4.

28 Obituary: Irina Press, *Telegraph* (31 May 2004) available at: http://www.telegraph.co.uk/ [accessed June 2010].

29 R. Tucker, The science and management of sex verification in sport, *South African Journal of Sports Medicine***21** (2009), pp. 147–50.

30 R. Ritchie, J. Reynard, T. Lewis, Intersex and the Olympic Games, *Journal of the Royal Society of Medicine***101** (2008), pp. 395–9.

31 S.L. Cavanagh, Transexual bodies at the Olympics, *Body & Society***12** (2006), pp. 75–102.

32 S Berg, How Dora the Man Competed in the Woman's High Jump, *Der Speigel International Online*http://www.spiegel.de/international/germany/0,1518,649104,00.html, dateline 15 Sep. 2009 [accessed July 2010].

33 Including the apparently widespread use (or alleged use) of hormone treatments in professional and amateur sport in the UK – see the use of ‘glands’ by Wolverhampton Wanderer football clubs in V. Heggie, A History of British Sports Medicine, Manchester University Press, Manchester (2011), Chapter Three.

